# Effects of the Kampo Formula Tokishakuyakusan on Headaches and Concomitant Depression in Middle-Aged Women

**DOI:** 10.1155/2014/593560

**Published:** 2014-02-04

**Authors:** Masakazu Terauchi, Shiro Hiramitsu, Mihoko Akiyoshi, Yoko Owa, Kiyoko Kato, Satoshi Obayashi, Eisuke Matsushima, Toshiro Kubota

**Affiliations:** ^1^Department of Women's Health, Tokyo Medical and Dental University, Yushima 1-5-45, Bunkyo, Tokyo 113-8510, Japan; ^2^Department of Obstetrics and Gynecology, Tokyo Medical and Dental University, Yushima 1-5-45, Bunkyo, Tokyo 113-8510, Japan; ^3^Department of Psychosomatics, Tokyo Medical and Dental University, Yushima 1-5-45, Bunkyo, Tokyo 113-8510, Japan

## Abstract

*Objectives*. To identify the correlates of headaches in middle-aged women and investigate the effects of *Tokishakuyakusan* (TJ-23), a formula of traditional Japanese herbal therapy *Kampo*, on headache and concomitant depression. *Methods*. We examined cross-sectionally the baseline records of 345 women aged 40–59 years who visited our menopause clinic. Among them, 37 women with headaches were treated with either hormone therapy (HT) or TJ-23; the data of these women were retrospectively analyzed to compare the effects of the treatment. *Results*. The women were classified into 4 groups on the basis of their headache frequency, and no significant intergroup differences were noted in the physical or lifestyle factors, except age. Multiple logistic regression analysis revealed that the significant contributors to the women's headaches were their age (adjusted OR 0.92 (95% CI 0.88–0.97)) and their depressive symptoms (adjusted OR 1.73 (95% CI 1.39–2.16)). Compared to women treated with HT, women treated with TJ-23 reported relief from headaches (65% versus 29%) and concomitant depression (60% versus 24%) more frequently. Improvement in the scores of headaches and depression correlated significantly with TJ-23 treatment. *Conclusions*. Headache in middle-aged women is significantly associated with depression; TJ-23 could be effective for treating both of these symptoms.

## 1. Introduction

Headache is one of the most common symptoms observed in community and primary care settings, as exemplified by a study revealing that 40% of the Nordic women in rural communities experience it, indicating that it is more frequent than other somatic symptoms [1]. As expected, headache is included in most of the inventories of menopausal symptom checklists, although the influence of menopause on women's headache depends on the subtypes of headache, such as tension-type headache (TTH) and migraine, which are the 2 most common ones. More than two-thirds of women with TTH report unchanged or worse status of headache after menopause [2]; on the other hand, migraine improves after menopause, and one of the triggers of migraine is postulated to be withdrawal of estrogen [3]. The 2 major types of primary headache mentioned above are, however, often difficult to differentiate in clinical practice and are summarized simply as “headaches” in most of the menopause assessment scales, such as Greene Climacteric Scale [4] and Women's Health Questionnaire [5].

Headache has been reported to be associated with psychological symptoms. A large-scale study on the Nordic community revealed that depression and anxiety were significantly associated with migraine and nonmigrainous headache and that the former was more strongly associated with psychological symptoms than the latter [6]. A correlation between headache and depression in middle-aged women was also noted in studies conducted in the United States [7] and Japan [8].

Kampo, a Japanese subdivision of traditional East Asian medicines, was founded in the 6th century as a local adaptation of the Chinese herbal medicine. Kampo is a more pragmatic approach to complementary and alternative medicine than the Chinese herbal medicine. In the Chinese herbal medicine, a medical practitioner confirms the treatment principle according to “Zheng” (the clinical diagnosis determined on the basis of an analysis of the patient's history, symptoms, and signs) and then mixes 10–15 herbs to create a specific formula for each patient. In Kampo, a practitioner chooses the best formula for a patient from approximately 150 ready-to-use formulae—each of which is typically composed of 5–9 herbs—on the basis of the patient's “Sho” (pattern of symptoms) [9, 10].

One of the most frequently used Kampo formulae in the management of menopausal symptoms in Japan is Tokishakuyakusan (TJ-23), which is an extract of granules made from a herb mixture of *Peony* root, *Atractylodes lancea* rhizome, *Alisma* rhizome, *Poria sclerotium*, and *Cnidium officinale* rhizome and the Japanese *Angelica *root. It is indicated for various symptoms, including headache, in patients who have generally weak muscles, are easily fatigued, and whose waist and lower limbs are susceptible to cold.

In the present study, we sought to determine the prevalence of headache and its correlates in the Japanese peri- and postmenopausal women and investigate the effect of the Kampo formula TJ-23 on their headache and concomitant depression.

## 2. Materials and Methods

In this retrospective study, we examined the medical records of the same study population examined in our previous study [8, 11–16]. Specifically, we analyzed the records of 345 Japanese peri- and postmenopausal women (age: 40–59 years) who had been enrolled in the Systematic Health and Nutrition Education Program (SHNEP) conducted at the Menopause Clinic of the Tokyo Medical and Dental University Hospital between February 2006 and December 2010. All the middle-aged women enrolled in this program had been referred to our clinic for the treatment of their menopausal symptoms and had provided informed consent for participation. Before beginning our investigation, we obtained approval for the study protocol from the Tokyo Medical and Dental University Review Board. All procedures in the study were in accordance with the Declaration of Helsinki.

The goals of SHNEP were to improve the general health status by prescribing appropriate medications after a physician's assessment and providing advice on diet and exercise regimens after lifestyle assessment by nutritionists.

The mean ± SD age of the 345 participants was 50.9 ± 4.5 years. The participants were classified as being in menopause transition or as postmenopausal, on the basis of an analysis of their menstrual cycles. A woman was defined as being in menopause transition if she had had a menstrual period within the past 12 months but had missed a period or if she had irregular cycles in the past 3 months. Women were classified into the postmenopausal group in the absence of a menstrual period in the past 12 months [17]. At their initial visit, the women were interviewed by physicians and nutritionists and provided data on their menopausal symptoms and quality of life during the past month by answering the Menopausal Health-Related Quality of Life (MHR-QOL) questionnaire, which has been developed and validated at our clinic [8, 11–16]. The MHR-QOL questionnaire is a modification of the Women's Health Questionnaire developed by Hunter [5, 18] and contains 38 items scored on a 4-point or a binary scale, covering 4 major domains (physical health, mental health, life satisfaction, and social involvement) of a woman's health during menopausal transition.

The items used to assess the 2 domains of interest in the current study, namely, physical health and mental health, are shown in Table 1. As shown, the physical health domain comprised 9 items that assess somatic symptoms, including headaches and vasomotor symptoms, and the mental health domain comprised 12 items that assess depressed mood, anxiety and fears, and sleep disturbances. For convenience, the scoring system used in the present study is the reverse of that used for the QOL questionnaire in our clinical practice; that is, the higher the scores for the 9 evaluated items, the poorer the physical and mental functioning: 0-1 time a month, 0 point; 1-2 times a week, 1 point; 3-4 times per week, 2 points; and almost every day, 3 points.

Data were recorded on the following physical characteristics of the women enrolled in the SHNEP: height (cm); body weight (kg); body mass index (BMI), which was calculated as weight (kg)/height^2^ (m^2^); waist circumference (cm); hip circumference (cm); waist-hip ratio, which was calculated as waist circumference/hip circumference; body fat percentage (%); lean body mass (kg); and body water mass (kg), with the latter 3 being measured using the body composition analyzer MC-190EM (Tanita, Tokyo, Japan). Systolic and diastolic blood pressure, pulse rate, cardio-ankle vascular index (CAVI), as an indicator for atherosclerosis, and ankle-brachial index (ABI), as an indicator for peripheral vascular disease, were measured using VS-1000 (Fukuda Denshi, Tokyo, Japan) [19].

The following lifestyle characteristics were also assessed: amount of daily caffeinated beverage consumption (more than 3 cups, 1–3 cups, none), frequency of alcohol consumption (daily, sometimes, never), and the habit of smoking (more than 20 cigarettes per day, 1–20 cigarettes per day, none).

Among the 182 participants who reported having headaches once or more than once a week at their first visits, 37 were treated with either hormone therapy (HT, *N* = 17) or TJ-23 (*N* = 20); these 37 patients were selected for further analysis. Estrogens included in the HT regimens used for these participants were either 0.625 mg/day of conjugated estrogen (*N* = 12), 50 *μ*g/day of estradiol via a transdermal patch (*N* = 3), and 1.0 mg of oral micronized estradiol (*N* = 2). For the women with an intact uterus (*N* = 8), medroxyprogesterone acetate was used either continuously or sequentially. TJ-23 was prescribed in the form of extract granules (7.5 g/day) comprising *Peony* root (9.7%), *A. lancea* rhizome, *Alisma *rhizome, *P. sclerotium*, *C. officinale* rhizome (7.3%), and Japanese *Angelica* root (TJ-23, Tsumura, Tokyo). We excluded the following subjects from the analysis: (1) women who were prescribed nonsteroidal anti-inflammatory drugs or triptans to treat their headaches or migraines; (2) women who were prescribed medications other than HT or TJ-23 alone to treat menopausal symptoms; and (3) women who were prescribed any psychotropic drugs, such as antidepressants, anxiolytics, and hypnotics.

All statistical analyses were performed with SAS 9.2 (SAS Institute, Cary, NC, USA). We performed statistical analyses using one-way analysis of variance, Fisher's exact test, multiple logistic regression with stepwise variable selection procedure, unpaired *t*-test, and the Kruskal-Wallis test. Statistical significance was defined as *P* < 0.05.

## 3. Results

Of the 345 women studied, 160 (46.4%) were in menopause transition and 185 (53.6%) were postmenopausal.

The prevalence of each item in the physical and mental health domains of the MHR-QOL questionnaire among the evaluated women is shown in Table 1. The percentages of women who experienced headaches 0-1 time a month, 1-2 times a week, 3-4 times per week, or almost every day were 47.2%, 27.5%, 11.6%, and 13.6%, respectively. Headache ranked 13th among the 21 items, assessed according to the percentage of women who experienced the symptom more than once a week (52.8%).

First, we compared the baseline characteristics of the 4 groups of women classified on the basis of their headache frequency (Table 2). There were no significant intergroup differences in any of the physical or lifestyle factors, except age. The average age of the women who had headaches almost every day was significantly less (by almost 2 years) than those of the women who experienced the symptom once a month or less frequently (*P* < 0.05, Tukey's test). Additionally, no significant intergroup differences were noted in the ratio of the women in menopause transition to those who were postmenopausal.

Second, we examined the correlations between headaches and other symptoms, namely, vasomotor, depressive, anxious, and insomnia. The scores for the 2 vasomotor symptoms in the MHR-QOL questionnaire (hot flushes and night sweats) were averaged to generate a vasomotor score. Likewise, the depression score, anxiety score, and insomnia score were calculated from the averages of the scores of 4 depressive symptoms (“loss of interest in things,” “lack of enjoyment,” “low energy,” and “depressed mood”), 2 anxious symptoms (“frightened/panicky feelings” and “feel tense/wound up”), and 2 insomnia symptoms scores (“difficulty in initiating sleep” and “nonrestorative sleep”), respectively. To determine whether these symptoms were associated with headaches in middle-aged women, we performed a multiple logistic regression analysis by using the presence of headaches once a week or more as the dependent variable and age and the scores for vasomotor symptoms, depression, anxiety, and insomnia as independent variables.  Table 3 shows the crude and adjusted ORs for assessing the strength of the relationship between each factor and headaches. Although all the variables included were significantly associated with headaches in the univariate logistic regression analysis, subsequent multiple logistic regression analysis with stepwise variable selection procedure revealed that only age (adjusted OR, 0.92; 95% CI, 0.88–0.97; *P* = 0.0019) and depression (adjusted OR, 1.73; 95% CI, 1.39–2.16; *P* < 0.0001) were significantly associated with headaches after adjustment. The highest condition index of 1.04 suggested the absence of collinearity among the 2 variables.

The baseline characteristics of the 37 study subjects treated with HT (*N* = 17) or TJ-23 (*N* = 20) for headaches are shown in Table 4. There were no significant differences between the groups regarding the age, headache frequency, or baseline symptom scores.

**Figure 1:**
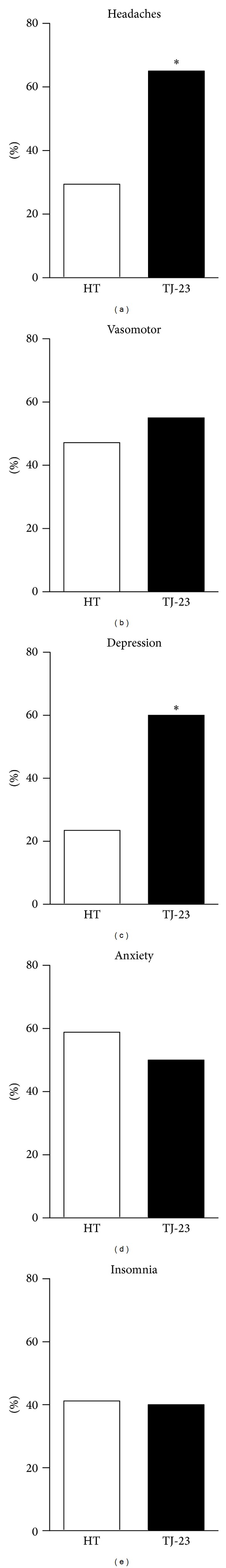
The percentage of women whose symptom scores decreased after treatment with hormone therapy (HT) or Tokishakuyakusan (TJ-23) *;  *P* < 0.05 versus HT.

The percentages of women whose symptom scores decreased after a follow-up period of 147 ± 56 (mean ± SD) days were compared. Significantly more women in the TJ-23 group reported relief from headaches and depression than those in the HT group (headaches, 65% versus 29%; depression, 60% versus 24%); however, there were no significant intergroup differences in the improvement of vasomotor symptoms, anxiety, and insomnia (Figure 1).

**Figure 2:**
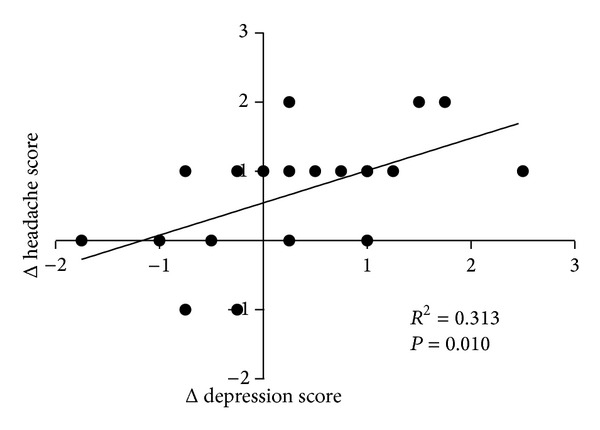
The association between the improvements in headaches and depression in the TJ-23 group.

Finally, an analysis of the association between improvement in headaches and depression in the TJ-23 group showed a significant correlation between the changes in the headache and depression scores with TJ-23 treatment (Figure 2).

## 4. Discussion

In this study, we aimed to identify the correlates of headaches in middle-aged women on the basis of a cross-sectional analysis of the records of 345 women participating in a health and education program. We also performed a retrospective cohort analysis to compare the effects of HT and the Kampo formula TJ-23 on the headaches and concomitant depression in these women.

A comparison of the physical and lifestyle factors among the groups of participants classified by their headache frequency suggested that younger women had more headaches than those who are older and that the symptom was not necessarily more frequent among women in menopause transition than in those who are postmenopausal. A classic article by Neugarten and Kraines [20] on menopausal symptoms indicated that the percentage of headaches in women aged over 55 years was remarkably lesser than that in women aged under 44 years, although the effect of menopause is difficult to ascertain because the study compared “menopausal” women with “pre- or postmenopausal” ones belonging to the same age group of 45–54 years. Recently, Berecki-Gisolf closely dissected the associations of age and menopause with the symptoms of middle-aged women in a large-scale prospective cohort study; they reported that age and postmenopause status, rather than perimenopause or menopause transition and premenopause, are significant negative contributors to headaches [21]. In our study, slightly more perimenopausal women (47%) were present in the group with headache almost every day than in the group with headaches at a frequency of once a month or less (44%). A larger sample size may have afforded a statistically significant intergroup difference.

Headache is known to be associated with psychological symptoms and is included in several somatization screening measures, such as Patient Health Questionnaire (PHQ)-15, World Health Organization (WHO)-Social Security Disability (SSD), and Symptom Checklist (SCL)-12 [22]. In the current study, the presence of headaches once a week or more frequently was found to be associated with depression but not with vasomotor symptoms, anxiety, or insomnia after adjustment. The association between depression and headaches was also confirmed in our previous study [8] and a US population-based study of middle-aged women [7]. On the other hand, a large-scale community study in Norway (HUNT-II) revealed that the Hospital Anxiety and Depression scale scores were significantly associated with the presence of migraine and nonmigrainous headaches [6]. The findings of the Nordic study in which the correlation with headaches is stronger for anxiety than for depression are not consistent with our finding; this discrepancy may be partly attributable to the differences in the sample backgrounds, such as age, (≥20 versus 40–60 years), sex (both versus female only), ethnicity (Nordic versus Japanese), and settings (community versus clinical).

The effects of ovarian sex steroids and menopause on headaches appear to differ from the type of headache. TTH is slightly more prevalent in women than in men, and the average of the reported male-to-female ratios is 1.30 [2]. This difference is not recognized until children reach puberty, thereby suggesting the involvement of sex steroids in the pathogenesis of TTH [2], although one report suggests that TTH status remains unchanged or becomes worse in 70% of women with this type of headache after menopause [23]. The sex difference is more marked in the case of migraine: the cumulative lifetime incidence for women is 43%, while that for men is 18% [3]. Migraine is known to be affected by hormonal fluctuations, with the effect of estrogen withdrawal being predominant during the perimenopausal period [3]. With the absence of fluctuations in sex hormone levels, the percentage of women reporting migraine after menopause is reduced, as demonstrated by the Penn ovarian aging study [24]. Although HT should be theoretically effective for perimenopausal migraine controlling the hormonal fluctuation, the reports are inconsistent. For example, a large population-based study showed a significant association between migraine and nonmigrainous headache with the current use of systemic HT [25]. In the present study, the percentages of women receiving HT whose headache improved, did not change, or worsened were 29%, 59%, and 12%, respectively, which were even better than the percentages indicated in Mueller's report on migraineurs only [26].

Complementary and alternative medicines (CAM) are extensively used worldwide, especially by women, middle-aged individuals, and people with chronic diseases or poor overall health [27]. In the early 2000s, approximately 50% of all middle-aged women in Western countries used CAM to alleviate menopausal symptoms [27, 28]. The current percentage may be even higher, considering the sustained decline in HT use among this population since the publication of the Women's Health Initiative results [29, 30]. A recent review by Adams revealed that a substantial proportion of people with headache and migraine use CAM and find it effective [31].

TJ-23 is one of the most frequently used Kampo formulae for the management of menopausal symptoms in Japan. According to the package insert, TJ-23 is indicated for the relief of climacteric disturbance (dull headache, headache, dizziness, shoulder stiffness, etc.) of patients who have generally weak muscles, are easily fatigued, and whose waist and lower limbs are susceptible to cold. The extracts of *Ligusticum chuanxiong*, a species closely related to the TJ-23 component *Cnidium officinale*, have long been used as an analgesic and were shown to alleviate headache [32] and migraine [33] in animal models. Recent reports also indicate that the Chinese equivalent of TJ-23, Danggui-Shaoyao-San, exerts antidepressant effects in animal models possibly by suppressing the expression of arginine vasopressin in the pituitary and hypothalamus [34]. The improvements in both headache and depression induced by TJ-23 could also be attributed to these pharmacological mechanisms. Furthermore, considering the association between headache and depression, it appears that the relieving effect of TJ-23 on headache may have helped improve the women's mood and vice versa [8].

One of the major limitations of our study is that our questionnaire and the subsequent analysis did not differentiate among the headache subtypes. Headaches have been clearly categorized by the International Headache Society as per the International Headache Classification system, with the intention of developing evidence-based treatment strategies for the patients. However, the accurate categorization of the headache subtype is sometimes difficult in clinical practice and may not be as useful to treatment as the proper assessment of the severity of the condition [35]. Furthermore, the retrospective design of the current study might have introduced a disparity between the treatment groups although no significant intergroup differences were observed in the background characteristics. Finally, further prospective studies worldwide using TJ-23 or Tokishakuyakusan are warranted to confirm the findings obtained from the current retrospective analysis of the limited number of subjects.

## 5. Conclusions

In conclusion, headaches in middle-aged women are significantly associated with depression. The Kampo formula TJ-23 could be an effective treatment for the women with both symptoms.

## Figures and Tables

**Table 1:** Prevalence of each item in the physical and mental health domains in the Menopausal Health Related Quality of Life (MHR-QOL) questionnaire (%, *N* = 345).

	0-1 time a month	1-2 times a week	3-4 times per week	Almost every day
Physical health domain				
Nausea	78.5	13.4	2.3	5.8
Dizziness	65.4	22.2	5.2	7.3
Numbness	60.4	13.5	6.7	19.4
Muscle and joint pains	12.8	18.6	13.3	55.4
Tiredness	14.5	21.2	15.1	49.3
Headaches	47.2	27.5	11.6	13.6
Frequent urination	52.5	15.2	8.2	24.2
Hot flushes	45.3	18.0	15.1	21.5
Night sweats	55.4	17.4	10.4	16.8
Mental health domain				
Loss of interest in things	51.9	18.1	13.1	16.9
Lack of enjoyment	48.7	22.0	11.9	17.4
Low energy	34.9	27.3	14.2	23.5
Depressed mood	30.8	27.6	15.7	25.9
Poor memory	27.5	32.5	20.6	19.4
Difficulty in concentration	31.6	29.3	20.3	18.8
Frightened/panicky feelings	46.2	22.7	15.4	15.7
Feel tense/wound up	37.5	24.1	17.2	21.2
Dissatisfaction with sexual relationship	85.0	6.8	2.9	5.3
Difficulty in initiating sleep	46.4	18.8	11.6	23.2
Nonrestorative sleep	34.5	21.4	13.6	30.4
Low self-esteem	39.1	21.0	12.0	28.0

**Table 2:** Baseline characteristics of the study participants by groups classified on the basis of their headache frequency (*N* = 345).

	0-1 time a month (*N* = 163)	1-2 times a week (*N* = 95)	3-4 times per week (*N* = 40)	Almost every day (*N* = 47)	*P* value
Age (years)	51.6 ± 4.5	50.1 ± 4.3	51.1 ± 4.7	49.7 ± 4.1	0.012
Menopause status (%)					
Menopause transition	44	48	53	47	0.737
Postmenopausal	56	52	48	53	
Height (cm)	156.2 ± 10.9	158.0 ± 4.6	156.7 ± 4.8	156.7 ± 4.6	0.406
Body weight (kg)	52.8 ± 8.1	53.0 ± 7.9	53.2 ± 11.3	54.3 ± 10.7	0.809
Body mass index (kg·m^−2^)	21.5 ± 3.3	21.3 ± 3.1	21.6 ± 3.6	22.1 ± 4.2	0.639
Waist circumference (cm)	77.9 ± 9.3	77.4 ± 8.5	79.0 ± 10.9	78.4 ± 11.4	0.824
Hip circumference (cm)	91.4 ± 6.1	90.8 ± 5.5	91.3 ± 7.8	91.9 ± 7.5	0.819
Waist-hip ratio	0.85 ± 0.06	0.85 ± 0.06	0.86 ± 0.07	0.85 ± 0.07	0.771
Body fat (%)	26.7 ± 7.4	27.0 ± 7.9	28.5 ± 7.9	27.0 ± 8.9	0.733
Muscle mass (kg)	35.6 ± 2.8	36.1 ± 2.8	36.0 ± 3.1	36.0 ± 2.8	0.648
Body water mass (kg)	27.2 ± 2.8	27.4 ± 2.9	27.6 ± 3.1	27.6 ± 3.1	0.885
Systolic pressure (mmHg)	124.3 ± 16.5	122.5 ± 12.4	127.7 ± 19.3	123.8 ± 14.1	0.382
Diastolic pressure (mmHg)	79.9 ± 11.4	79.0 ± 8.8	80.9 ± 12.4	79.3 ± 11.6	0.801
Pulse rate (min^−1^)	62.7 ± 10.4	61.0 ± 12.2	62.2 ± 8.2	64.6 ± 10.2	0.297
Cardio-ankle vascular index (CAVI)	7.54 ± 0.68	7.40 ± 0.60	7.48 ± 0.75	7.55 ± 0.74	0.479
Ankle-brachial index (ABI)	1.11 ± 0.06	1.12 ± 0.06	1.11 ± 0.06	1.11 ± 0.07	0.930
Alcohol consumption (%)					
Daily	15	10	11	9	0.711
Sometimes	22	30	26	22	
None	63	60	63	69	
Smoking (%)					
≥20 cigarettes/day	4	3	5	7	0.821
<20 cigarettes/day	9	12	5	11	
None	87	85	89	82	
Caffeinated beverage consumption (%)					
≥3 cups/day	70	68	68	53	0.365
<3 cups/day	22	23	29	38	
None	8	9	3	9	

Data are expressed as the mean ± standard deviation or percentage.

The *P* values were derived from one-way analysis of variance or Fisher's exact test.

**Table 3:** Contribution of age, vasomotor symptoms, depression, anxiety, and insomnia to headaches in peri- and postmenopausal women (*N* = 345).

	Crude OR (95% CI)	*P* value	Adjusted OR (95% CI)	*P* value
Age	0.93 (0.89–0.98)	0.0038	0.92 (0.88–0.97)	0.0019
Vasomotor score	1.31 (1.06–1.63)	0.0136		
Depression score	1.69 (1.36–2.10)	<0.0001	1.73 (1.39–2.16)	<0.0001
Anxiety score	1.51 (1.23–1.86)	<0.0001		
Insomnia score	1.40 (1.16–1.70)	0.0006		

OR: odds ratio; CI: confidence interval.

**Table 4:** Baseline characteristics of the study subjects (*N* = 37).

	HT (*n* = 17)	TJ-23 (*n* = 20)	*P* value
Age (years)	48.9 (3.6)	50.7 (5.4)	0.249
Frequency of headaches (%)			
1-2 times a week	71	50	0.531
3-4 times per week	18	25	
Almost every day	12	25	
Vasomotor score	1.26 (1.16)	1.38 (1.19)	0.756
Depression score	1.24 (0.68)	1.54 (0.75)	0.124
Anxiety score	1.29 (1.05)	1.48 (0.85)	0.439
Insomnia score	1.56 (1.16)	1.70 (1.20)	0.731

Data are expressed as the mean (standard deviation) or percentage.

*P* values were derived from the unpaired *t-*test (age), Fisher's exact test (frequency of headaches), or Kruskal-Wallis test (symptom scores).
